# Cognitive performance in individuals aged 80 or over from south of Brazil

**DOI:** 10.1002/alz.092303

**Published:** 2025-01-03

**Authors:** Josiane Budni, Gabriela Serafim Keller, Ana Carolina Brunatto Falchetti Campos, Eduarda Behenck Medeiros, Adrielly Vargas Lídio, Luísa Rosler Grings, Débora Dagostin Casagrande, Laura Ceolin De Jesus, Gabriela Piovesan Fenilli, Amanda L Boaventura, Gabriel Casagrande Zabot, Luciane B Ceretta

**Affiliations:** ^1^ University of Southern Santa Catarina (UNESC), Criciuma, SC Brazil

## Abstract

**Background:**

The population aged 80 or over has increased, which may predispose people to dementia. Validated scales are used to diagnose them. The study aimed to compare cognitive evolution and associated factors in individuals aged 80 or over.

**Method:**

The Research Ethics Committee from the University of Southern Santa Catarina (UNESC) authorized the study under 3,214,698. It was an observational, quantitative study with a cohort design. Individuals aged 80 or over were recruited for this study. One hundred twenty‐four participants were evaluated in 2016. After six years, they were reevaluated, with 102 participants lost to follow‐up, resulting in 22 participants for reapplication. In addition, 78 new participants entered the study, totaling 100 participants in 2022. Cognitive tests such as the Mini‐Mental State Examination (MMSE), Clock Test, Verbal Fluency, and Montreal Cognitive Assessment (MOCA) were applied.

**Result:**

When comparing the two times, there was a significant difference in the percentage of retirement and participation in some activities in the community. Compared to cognitive tests, a decrease in cognitive decline and verbal fluency was observed in 2022, assessed by the MMSE. When the 22 individuals were evaluated over six years, there was a significant change in participation in community activities. When asked about difficulty remembering things, people, or situations, there was a reduction in 2022. Unlike the MMSE and the verbal fluency test, the MOCA showed cognitive impairment in most individuals in 2022.

**Conclusion:**

It is concluded that the population of 2016 and 2022 showed some differences related to cognition, but this difference was not observed in the 22 individuals. These results indicate a divergence between the tests. Considering the many differences in the Brazilian population, it is necessary to standardize a cognitive test suitable for screening cognitive decline.

